# Supplementary material for: Identifying Differences in Consumer Attitudes towards Local Foods in Organic and National Voluntary Quality Certification Schemes
Source: Foods. 2023 Mar 8;12(6):1132. doi: 10.3390/foods12061132 (PMC10048378; doi:10.3390/foods12061132)
Supplement: Supplementary file 1 [file foods-12-01132-s001.zip › foods-2248648-supplementary.pdf]

Supplementary materials

**Table S1.** Cronbach's Alpha Measures Internal Consistency and Exploratory Factor Analysis Determining Latent Variables' Validity: Factor Loading and Means (*n* = 1007).

| **Measuring Items** | **Factor Loadings** | |
|---|---|---|
| | **Factor 1** | **Factor 2** |
| **Interest in information cues denoting quality, traceability, sustainability, and origin (Cronbach's alpha = 0.76; Variance explained = 40.8%)** | | |
| *When shopping, I check the ingredients list on a regular basis.* | 0.854 | |
| *When shopping, I check the nutrition facts on a regular basis.* | 0.729 | |
| *When shopping, I check the geographical origin on a regular basis.* | 0.589 | |
| *When shopping, I check the shelf life on a regular basis.* | 0.313 | |
| *When shopping, I check the sustainability aspect of the food, especially the environmental impact.* | 0.581 | |
| **Understanding the definition of local food (Cronbach's alpha = 0.46; Total variance explained = 53.3%)** | | |
| Regional-based interpretation (Variance explained = 32.3%) | | |
| *Local foods are those that are produced in the region where consumers live.* | 0.198 | |
| *Local foods are only those that can be purchased from a farm.* | 0.267 | |
| *Local foods are those produced by the traditional methods recognized in the area where they are sold.* | 0.865 | |
| Country-based interpretation (Variance explained = 21.0%) | | |
| *Local foods are those that are produced in the country where consumers live.* | | 0.398 |
| *Local foods are those produced by all the farms and the food industry in the country.* | | 0.696 |
| **Trust in actors of local food production (Cronbach's alpha = 0.79; Total variance explained = 67.2%)** | | |
| Person-related trust dimension (Variance explained = 39.6%) | | |
| *I trust the local farmers.* | 0.963 | |
| *I trust the Slovenian farmers.* | 0.813 | |
| Institution-/Corporation-related trust dimension (Variance explained = 27.6%) | | |
| *I trust food manufacturers that indicate local origin.* | | 0.716 |
| *I trust food retailers' labels that indicate local origin.* | | 0.710 |
| *I trust the authorities at the national and EU level that monitor compliance with local production standards.* | | 0.584 |
| **Local food perception (Cronbach's alpha = 0.84; Variance explained = 48.7%)** | | |
| Lionization and opposition (Variance explained = 39.8%) | | |
| *Locally produced foods just taste better.* | 0.590 | |
| *Locally produced foods are more nutritious than foods that have been shipped from somewhere else.* | 0.471 | |
| *Buying locally produced foods supports sustainable farming practices.* | 0.743 | |
| *Buying local foods helps build a more prosperous community and rural development.* | 0.795 | |
| *I like to support local farmers and local manufacturers whenever possible.* | 0.668 | |
| *Supporting the local food economy is important to me.* | 0.709 | |
| Communalization (Variance explained = 8.9%) | | |
| *I don't trust foods that have been produced by large, multinational corporations.* | | 0.586 |

| | |
|---|---|
| *I would go out of my way to avoid buying food from a large retail grocery chain.* | 0.707 |
| *I feel uneasy eating something unless I know exactly where it was produced.* | 0.540 |

Note. Extraction Method: Maximum Likelihood. Rotation Method: Varimax with Kaiser Normalization.

**Table S2.** Sample and Groups Mean (SD) for Latent Variables' Measuring Items.

| Latent Variables' Measuring Items | Nationally Representative Sample | "I Trust the Organic Label" | "I Trust the Selected Quality Label" |
|---|---|---|---|
| **Items indicating interest in information cues denoting quality, traceability, sustainability, origin and intention towards purchasing local food.*** | 4.58 (1.18) | 4.74 (1.24) | 4.64 (1.20) |
| *When shopping, I check the ingredients list on a regular basis.* | 4.38 (1.71) | 4.41 (1.75) | 4.36 (1.70) |
| *When shopping, I check the nutrition facts on a regular basis.* | 3.96 (1.78) | 3.99 (1.94) | 3.89 (1.72) |
| *When shopping, I check the geographical origin on a regular basis.* | 4.65 [a] (1.68) | 4.94 (1.53) | 4.90 (1.79) |
| *When shopping, I check the shelf life on a regular basis.* | 5.91 (1.43) | 6.04 (1.18) | 6.05 (1.37) |
| *When shopping, I check the sustainability aspect of the food, especially the environmental impact.* | 4.00 (1.64) | 4.16 (1.75) | 4.13 (1.72) |
| *The next time I buy food, I will pay attention to local food.* | 5.47 (1.42) | 5.63 (1.40) | 5.80 (1.11) |
| **Understanding the definition of local food** | | | |
| Regional-based interpretation (3 items)* | 4.84 (1.19) | 4.79 (1.25) | 4.99 (1.12) |
| *Local foods are those that are produced in the region where consumers live.* | 5.61 (1.62) | 5.35 (1.75) | 5.67 (1.45) |
| *Local foods are only those that can be purchased from a farm.* | 4.12 (2.02) | 4.09 (2.02) | 4.33 (2.07) |
| *Local foods are those produced by the traditional methods recognized in the area where they are sold.* | 4.81 (1.72) | 4.93 (1.75) | 4.97 (1.75) |
| Country-based interpretation (2 items)* | 4.24 (1.57) | 4.21 (1.53) | 4.15 (1.50) |
| *Local foods are those that are produced in the country where consumers live.* | 4.48 (2.04) | 4.43 (2.04) | 4.53 (2.01) |
| *Local foods are those produced by all the farms and the food industry in the country*. | 4.0 (1.87) | 4.00 (1.79) | 3.77 (1.94) |
| **Attitudes towards local food** | | | |
| Lionization and opposition (6 items)* | 5.52 (1.10) | 5.58 (1.10) | 5.75 (0.98) |
| *Locally produced foods just taste better.* | 5.42 (1.48) | 5.49 (1.45) | 5.55 (1.49) |
| *Locally produced foods are more nutritious than foods that have been shipped from somewhere else.* | 5.26 (1.72) | 5.25 (1.77) | 5.36 (1.73) |
| *Buying locally produced foods supports sustainable farming practices.* | 5.64 (1.38) | 5.62 (1.38) | 5.88 (1.18) |
| *Buying local foods helps build a more prosperous community and rural development.* | 5.93 (1.29) | 5.72 (1.39) | 6.17 (1.11) |
| *I like to support local farmers and local manufacturers whenever possible.* | 5.40 (1.44) | 5.56 (1.36) | 5.74 (1.35) |
| *Supporting the local food economy is important to me.* | 5.48 (1.41) | 5.58 (1.31) | 5.78 (1.28) |
| Communalization (3 items)* | 4.28 (1.37) | 4.52 (1.30) | 4.66 (1.38) |
| *I don't trust foods that have been produced by large, multinational corporations.* | 4.78 (1.72) | 5.05 (1.72) | 5.15 (1.69) |
| *I would go out of my way to avoid buying food from a large retail grocery chain.* | 3.82 (1.66) | 3.82 (1.51) | 4.09 (1.63) |

| | | | |
|---|---|---|---|
| *I feel uneasy eating something unless I know exactly where it was produced.* | 4.25 (1.89) | 4.52 (1.73) | 4.65 (1.78) |
| **Trust in actors producing local food** | | | |
| Person-related dimension (2 items)* | 5.50 (1.23) | 5.50 (1.16) | 5.71 (1.15) |
| *I trust the local farmers.* | 5.58 (1.30) | 5.55 (1.22) | 5.80 (1.18) |
| *I trust the Slovenian farmers.* | 5.41 (1.31) | 5.35 (1.23) | 5.67 (1.28) |
| Institution-/Corporation-related dimension (3 items)* | 4.47 (1.22) | 5.50 (1.16) | 5.71 (1.15) |
| *I trust food manufacturers that indicate local origin.* | 4.52 (1.42) | 4.54 (1.26) | 4.40 (1.20) |
| *I trust food retailers' labels that indicate local origin.* | 4.46 (1.43) | 4.47 (1.46) | 4.42 (1.34) |
| *I trust the authorities at the national and EU level that monitor compliance with local production standards.* | 4.44 (1.49) | 4.53 (1.52) | 4.35 (1.40) |
| **Willingness to purchase local food*** | 5.54 (1.23) | 5.90 (1.11) | 5.91 (0.99) |
| *How likely is it that you will purchase local foods?* | 5.62 (1.27) | 5.90 (1.16) | 5.93 (1.05) |
| *How likely is it that you will buy your basic food items from a neighborhood grocery store that offers locally produced food items?* | 5.46 (1.35) | 5.80 (1.17) | 5.89 (1.08) |

Note. * Corresponding dimensions and number of items of latent variables (bold). The individual scores of latent variables and corresponding dimensions were calculated based on the sum of responses on all items divided by the number of items.

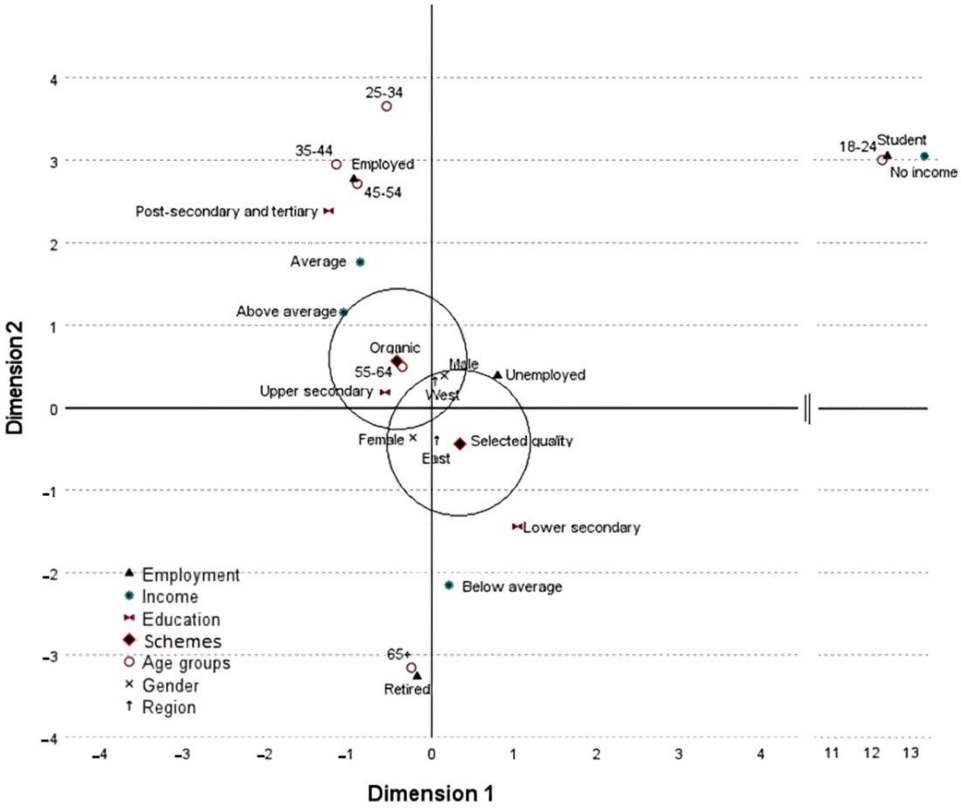


**Figure S1.** Graphical Display in Two Dimensions of 23 Categories Only in Principal Coordinates, Resulting from the Joint Correspondence Analysis (JCA).

**Table S3.** Attribute Importance and Contribution of Slovenian and Local Food in Purchased Food Product Categories.

| **Variable** | **Cereal and Cereal Products (*n* = 206)** | | **Milk and Dairy Products (*n* = 201)** | | **Meat and Meat Products (*n* = 196)** | | **Vegetable Products (*n* = 226)** | | **Fruit Products (*n* = 178)** | |
|---|---|---|---|---|---|---|---|---|---|---|
| **Attribute importance** (multiple choice) | ***n* (%)** | **Rank** | ***n* (%)** | **Rank** | ***n* (%)** | **Rank** | ***n* (%)** | **Rank** | ***n* (%)** | **Rank** |
| *Produced locally* | 114 (16.5) | 1 | 90 (13.2) | 3 | 107 (15.8) | 2 | 161 (19.4) | 1 | 114 (18.8) | 1 |
| *Country of origin (COO)* | 92 (13.3) | 3 | 102 (14.9) | 2 | 100 (14.8) | 3 | 113 (13.6) | 3 | 89 (14.7) | 3 |
| *Ingredients' origin traceability* | 39 (5.6) | 9 | 53 (7.8) | 8 | 40 (5.9) | 8 | 33 (4.0) | 8 | 28 (4.6) | 8 |
| *Traditional recipe* | 41 (5.9) | 8 | 29 (4.2) | 9 | 26 (3.8) | 19 | 35 (4.2) | 9 | 12 (2.0) | 10 |
| *Seasonal food* | 39 (5.6) | 9 | 23 (3.4) | 10 | 30 (4.4) | 9 | 126 (15.2) | 2 | 107 (17.7) | 2 |
| *Sustainable production* | 83 (12.0) | 4 | 60 (8.8) | 7 | 62 (9.2) | 6 | 83 (10.0) | 6 | 56 (9.3) | 6 |
| *Animal welfare* | 44 (6.4) | 7 | 66 (9.7) | 5 | 71 (10.5) | 4 | 31 (3.7) | 10 | 24 (4.0) | 9 |
| *Quality control* | 102 (14.8) | 2 | 113 (16.5) | 1 | 111 (16.4) | 1 | 97 (11.7) | 4 | 69 (11.4) | 4 |
| *Farm produced* | 58 (8.4) | 6 | 64 (9.4) | 6 | 61 (9.0) | 7 | 89 (10.7) | 5 | 57 (9.4) | 5 |
| *Ingredients' origin traceability* | 79 (11.4) | 5 | 83 (12.2) | 4 | 68 (10.1) | 5 | 63 (7.6) | 7 | 49 (8.1) | 7 |
| **Contribution of Slovenian origin products in usual food category purchases:** mean % (*SD*) | 63.4 (25.6) | | 77.2 (24.1) | | 64.8 (26.4) | | 56.8 (22.6) | | 55.8 (22.2) | |
| **Contribution of local products in Slovenian origin products in usual food category purchases:** mean % (*SD*) | 36.2 (26.5) | | 38.0 (30.9) | | 40.1 (27.3) | | 37.9 (24.5) | | 32.1 (24.1)) | |
